# Racial Variations in Retinal Oxygen Metabolic Biomarkers in Diabetic Retinopathy: A Pilot Study

**DOI:** 10.1155/joph/5621029

**Published:** 2026-05-05

**Authors:** Katherine Tavasoli, Sophie Leahy, Norman P. Blair, Farzan Abdolahi, Anthony E. Felder, Mahnaz Shahidi

**Affiliations:** ^1^ Keck School of Medicine, University of Southern California, Los Angeles, California, USA, usc.edu; ^2^ Department of Ophthalmology, University of Southern California, Los Angeles, California, USA, usc.edu; ^3^ Department of Ophthalmology and Visual Sciences, University of Illinois at Chicago, Chicago, Illinois, USA, uic.edu; ^4^ Richard and Loan Hill Department of Biomedical Engineering, University of Illinois Chicago, Chicago, Illinois, USA, uic.edu; ^5^ Alfred E. Mann Department of Biomedical Engineering, University of Southern California, Los Angeles, California, USA, usc.edu

**Keywords:** diabetes, oxygen, race, retina

## Abstract

**Purpose:**

Previous studies have reported that race affects the prevalence and progression of diabetic retinopathy (DR). In this pilot study, we tested the hypothesis that there are associations between race and retinal oxygen metabolic biomarkers in subjects without diabetes or with diabetes at early stages of DR.

**Methods:**

A total of 104 subjects participated and were categorized into one of three diagnosis groups: nondiabetic (ND), diabetic without clinical DR (NDR), or diabetic with mild nonproliferative DR (mild NPDR). Subjects were also classified according to race as Asian (AS), African American (AA), Hispanic White (HW), or non‐HW (NHW). Retinal oxygen metabolic biomarkers, namely, inner retinal oxygen delivery (DO_2_), metabolism (MO_2_), and extraction fraction (OEF), were determined by multimodal imaging (oximetry and Doppler optical coherence tomography).

**Results:**

There were significant interactions of race and diagnosis group on retinal oxygen metabolic biomarkers. The effect of diagnosis group on MO_2_ was significant in the AS group, such that MO_2_ was lower in the NDR than in mild NPDR. The diagnosis group did not have a significant effect on DO_2_, OEF, or MO_2_ in other races. In the ND group, there was no significant effect of race on MO_2_, while DO_2_ was marginally higher in HW than NHW. OEF was higher in AS than in HW and NHW. In the NDR group, there was no significant effect of race on OEF or MO_2_, while DO_2_ was higher in AA than AS, HW, and NHW. In mild NPDR, MO_2_ and OEF were higher in AS than NHW, and DO_2_ was not significantly affected by race.

**Conclusion:**

The preliminary findings demonstrated associations of race with retinal oxygen metabolic biomarkers, underscoring the necessity for future studies in larger cohorts with the inclusion of potential confounding factors to establish normal baselines according to race that are needed for considering racial differences for clinical evaluation of DR.

## 1. Introduction

Diabetic retinopathy (DR) is a neurovascular complication of diabetes and one of the leading causes of moderate and severe vision loss worldwide. It is estimated that 9.6 million people in the United States have DR, and of those diagnosed, 1.84 million have vision‐threatening DR [1], making the condition a significant public health concern. DR is classified into two forms: nonproliferative (NPDR) and proliferative (PDR). The nonproliferative form is characterized by retinal microaneurysms, hemorrhages, and exudates [2] in the absence of neovascularization. Over time, degeneration of the retinal microvasculature in NPDR can cause vision loss via several mechanisms. Breakdown of endothelial tight junctions and subsequent increased vascular permeability result in leakage of vascular contents into the retina, known as macular edema. Macular edema is the most common cause of vision loss in patients with NPDR [3]. Another major complication of NPDR is nonperfusion of retinal capillaries. This occurs when capillary pericytes are lost, which causes the vessel to dilate, resulting in decreased resistance to flow within the affected capillary and nonperfusion of adjacent capillaries due to shunt formation [4]. Next to areas of nonperfusion, intraretinal microvascular abnormalities develop, which are associated with increasing nonperfusion and ischemia. In response to chronic ischemia, neovascularization occurs [5], defining the progression from NPDR to PDR. The most severe form of DR is PDR and can result in blindness. Therefore, identification of retinal physiological biomarkers for the detection and monitoring of DR is needed to improve clinical outcomes.

The prevalence of diabetes in the United States is greater in Hispanic Whites (HWs) (22.1%), African Americans (AAs) (20.4%), and Asians (ASs) (19.1%) than non‐HWs (NHWs) (12.1%) [6]. Consistent with this prevalence, HbA1c levels are also higher in HWs, AAs, and ASs than in NHWs [7]. In addition, race has been shown to affect both the prevalence [8–11] and treatment of DR [12, 13]. Importantly, AAs with diabetes are four times more likely to be visually impaired by DR than NHWs with diabetes [14]. Similarly, the probability of sight‐threatening DR is higher in AS compared to people in White racial groups with diabetes, even after adjusting for age and duration of diabetes [8]. Lastly, in people with diabetes, the prevalence of DR and macular edema was significantly higher in HWs (37.4% and 10.7%) than in NHWs (24.8% and 2.7%) [15].

Historically, NHWs have been overrepresented in clinical trials for diabetic macular edema and DR, while AAs have been underrepresented compared to their estimated racial disease burdens [16]. Underrepresentation of minority groups in clinical trials ultimately compromises the quality of their care, as standards of care are then developed based on a subset of the population that may not adequately represent all people. Overall, with the known racial differences in the prevalence of diabetes and development of DR, it is necessary to consider race in evaluating retinal pathophysiology.

Previous studies have reported the effect of race and pigmentation on retinal oxygen saturation of hemoglobin (SO_2_) measurements [17–19]. Using retinal vascular SO_2_ and blood flow measurements, retinal oxygen metabolic biomarkers, namely, rates of oxygen delivery by the inner retinal circulation (DO_2_), oxygen extracted by the inner retinal tissue for metabolism (MO_2_), and inner retinal oxygen extraction fraction (OEF) have been established [20–30]. In diabetes, reductions in MO_2_ have been reported in no or mild DR [31] and moderate to severe NPDR [32, 33]. Moreover, OEF was reduced in NPDR subjects compared to nondiabetics (NDs) [34]. In PDR, both MO_2_ and DO_2_ were shown to be reduced [33, 35]. However, the findings of most studies were based on NHW cohorts and did not account for racial differences, thus limiting their relevance to all diabetic populations. The purpose of the current study was to test the hypothesis that there is an association between race and retinal oxygen metabolic biomarkers in subjects without diabetes or with diabetes at early stages of DR.

## 2. Materials and Methods

### 2.1. Subjects

The research study was approved by the Institutional Review Board of the University of Southern California. Prior to enrollment, the purpose of the study was explained to each participant, and informed consent was obtained in accordance with the tenets of the Declaration of Helsinki. Subjects with Type 2 diabetes and a clinical diagnosis of no or mild NPDR according to the International Clinical DR Severity Scale [36] and subjects without diabetes were recruited. Exclusion criteria included opacities of the ocular media, fixation instability affecting image quality, myopia greater than 6 diopters, and ocular conditions such as retinal vascular occlusions, age‐related macular degeneration, and glaucoma. Patients with diagnosed systemic diseases that are known to affect the retina were excluded. After exclusion criteria, a total of 104 subjects participated in the study. The racial classification of subjects was self‐reported, with the following categories: East AS (*N* = 15), AA (*N* = 9), HW (*N* = 62), or NHW (*N* = 18). Data from a subset of these subjects were included in our previous publication [33]. Based on a comprehensive clinical examination, one eye of each subject was classified into one of three categories: ND control (*N* = 27), diabetic with no DR (NDR) (*N* = 44), or diabetic with mild NPDR (NPDR) (*N* = 33). Mean arterial pressure (MAP), intraocular pressure (IOP), hematocrit (HCT), and hemoglobin A1c (HbA1c) were measured. Ocular perfusion pressure (OPP) was determined using the formula: ⅔ (MAP – IOP). Prior to imaging, the pupils were dilated using 1% tropicamide and 2.5% phenylephrine.

### 2.2. Image Acquisition and Analysis

Multimodal imaging was performed by our previously described techniques [20, 23, 33]. In brief, total retinal blood flow (TRBF) was measured using a commercially available optical coherence tomography system (Avanti; Optovue Inc., Fremont, CA, USA). Volumetric images covering a 2  ×  2 mm area centered on the optic nerve head were analyzed using a customized phase‐resolved technique, as previously described [37]. Dual wavelength (570 nm and 606 nm) retinal oximetry was performed with the use of our custom‐built imaging system, as previously described [25]. Images covering a 5 × 5 mm area centered on the optic nerve head were generated for oximetry.

Previous studies have shown that measurements of retinal SO_2_ are affected by the level of retinal pigmentation [17, 19]. Therefore, SO_2_ values were corrected based on retinal pigmentation levels. In brief, an optical density ratio (ODR) was calculated based on the ratio of optical densities (log‐transformed ratio of intensities outside to inside of the vessel) from images at each of the two wavelengths [25]. For each eye, a pigmentation index was determined based on the ratio of the average pixel intensities outside the vessels from images at the two wavelengths [18]. Linear relationships of ODR with vessel diameter and pigmentation index were determined empirically and utilized to adjust the ODR values to eliminate artificial dependence on either determinant. Adjusted ODR values were converted to arterial and venous SO_2_ (SO_2A_ and SO_2V_) [25, 38].

Mean SO_2A_ and SO_2V_ values were converted to blood oxygen content (O_2A_ and O_2V_), respectively, using the oxygen‐binding capacity of hemoglobin [39] and the subject’s hemoglobin concentration calculated from the measured HCT value. DO_2_, MO_2_, and OEF were calculated using the following equations: TRBF × O_2A_, TRBF × (O_2A_–O_2V_), and MO_2_/DO_2_, respectively.

### 2.3. Statistical Analysis

Data were analyzed using SPSS Statistics Premium Version 29. The distributions of the retinal oxygen metabolic biomarkers (DO_2_, MO_2_, and OEF) were examined for data normality using the Shapiro–Wilk test. Chi‐squared tests and one‐way analysis of variance (ANOVA) were used to determine differences in the categorical variables (sex and diagnosis group) and continuous variables (age, HbA1c, MAP, IOP, and OPP) among the races, respectively. Univariate two‐way ANOVAs were used to evaluate the interaction effect of race (AA, AS, HW, and NHW) and the diagnosis group (ND, NDR, and mild NPDR) on retinal oxygen metabolic biomarkers (DO_2_, MO_2_, and OEF). Since race and diagnosis group interactions were significant, the simple main effects of race and the diagnosis group on the retinal oxygen metabolic biomarkers were tested by ANOVAs. The estimated effect size (*η* = partial eta squared) and observed power (OP) were determined. Post hoc (Tukey) pairwise comparisons were performed, accounting for multiple comparisons. All statistical tests were two‐tailed, and *p* ≤ 0.05 was considered significant.

## 3. Results

The demographic and clinical characteristics of the subjects stratified by race (AS, AA, HW, and NHW) are presented in Table 1. Age, sex, diagnosis group, HbA1c, MAP, IOP, and OPP were not significantly different among races (*p* ≥ 0.30). Since these demographic and systemic factors were not significantly different among races, their confounding effects were not accounted for in the statistical analyses.

**TABLE 1:** Demographic and clinical characteristics of subjects.

	**Total (*N* = 104)**	**AS (*N* = 15)**	**AA (*N* = 9)**	**HW (*N* = 62)**	**NHW (*N* = 18)**	**p** **value**

Age (years)	59 ± 12	60 ± 12	61 ± 13	59 ± 12	55 ± 13	0.54
Sex						0.64
Male	46	5	4	27	10	
Female	58	10	5	35	8	
Group						0.30
ND	27	8	2	13	4	
NDR	44	4	4	29	7	
Mild NPDR	33	3	3	20	7	
HbA1c	6.8 ± 1.2	6.5 ± 1.0	6.6 ± 1.0	6.8 ± 1.1	7.2 ± 1.7	0.34
MAP (mmHg)	100 ± 14	102 ± 14	102 ± 16	98 ± 13	102 ± 18	0.72
IOP (mmHg)	14.7 ± 3.2	14.8 ± 2.5	15.6 ± 3.5	14.3 ± 3.0	15.7 ± 4.1	0.33
OPP (mmHg)	56.7 ± 9.3	58 ± 9	58 ± 11	56 ± 9	57 ± 12	0.87

*Note:* Mean ± SD are shown.

Abbreviations: AA, African American; AS, Asian; HbA1c, glycosylated hemoglobin; HW, Hispanic White; IOP, intraocular pressure; MAP, mean arterial pressure; Mild NPDR, mild nonproliferative diabetic retinopathy; ND, nondiabetic; NDR, no diabetic retinopathy; NHW, non‐Hispanic White; OPP, ocular perfusion pressure.

### 3.1. Effect of the Diagnosis Group and Race Interactions

There were significant interactions of race and diagnosis group on retinal oxygen metabolic biomarkers (*p* ≤ 0.04). Therefore, the simple main effects of the diagnosis group (for each racial group) and race (for each diagnosis group) were evaluated. Tables 2, 3, and 4 show the mean and standard deviations (SDs) of MO_2_, DO_2_, and OEF stratified by diagnosis group and race.

**TABLE 2:** Inner retinal oxygen metabolism (MO_2_) by race and diagnosis group.

	**AS**	**AA**	**HW**	**NHW**	**p** **value**

ND	3.06 ± 0.91	2.95 ± 1.11	3.25 ± 1.05	1.85 ± 0.11	0.10
NDR	1.98 ± 0.82	3.63 ± 1.18	2.66 ± 0.99	2.62 ± 0.66	0.12
Mild NPDR	4.57 ± 1.87	2.28 ± 0.95	2.76 ± 1.47	1.72 ± 0.98	0.04
*p* value	0.03	0.34	0.33	0.09	

*Note:* Mean ± SDs of MO_2_ are shown. values of the effect of the diagnosis group (bottom row) and race (right column) by ANOVA are listed.

Abbreviations: AA, African American; AS, Asian; HW, Hispanic White; Mild NPDR, mild nonproliferative diabetic retinopathy; ND, nondiabetic; NDR, no diabetic retinopathy; NHW, non‐Hispanic White.

**TABLE 3:** Inner retinal oxygen delivery (DO_2_) by race and diagnosis group.

	**AS**	**AA**	**HW**	**NHW**	**p** **value**

ND	6.86 ± 1.58	7.42 ± 1.88	9.44 ± 3.11	5.78 ± 1.06	0.04
NDR	5.39 ± 1.00	10.74 ± 2.65	7.62 ± 2.17	6.79 ± 1.37	0.005
Mild NPDR	9.84 ± 4.35	5.71 ± 3.41	7.43 ± 3.05	5.98 ± 1.50	0.24
*p* value	0.06	0.13	0.08	0.42	

*Note:* Mean ± SDs of DO_2_ are shown. *p* values of the effect of the diagnosis group (bottom row) and race (right column) by ANOVA are listed.

Abbreviations: AA, African American; AS, Asian; HW, Hispanic White; Mild NPDR, mild nonproliferative diabetic retinopathy; ND, nondiabetic; NDR, no diabetic retinopathy; NHW, non‐Hispanic White.

**TABLE 4:** Inner retinal oxygen extraction fraction (OEF) by race and diagnosis group.

	**AS**	**AA**	**HW**	**NHW**	**p** **value**

ND	0.44 ± 0.05	0.39 ± 0.06	0.35 ± 0.05	0.32 ± 0.05	0.002
NDR	0.36 ± 0.11	0.34 ± 0.07	0.35 ± 0.08	0.39 ± 0.08	0.66
mild NPDR	0.49 ± 0.11	0.45 ± 0.12	0.36 ± 0.08	0.28 ± 0.12	0.01
p value	0.14	0.35	0.89	0.11	

*Note:* Mean ± SDs of OEF are shown. *p* values of the effect of the diagnosis group (bottom row) and race (right column) by ANOVA are listed.

Abbreviations: AA, African American; AS, Asian; HW, Hispanic White; Mild NPDR, mild nonproliferative diabetic retinopathy; ND, nondiabetic; NDR, no diabetic retinopathy; NHW, non‐Hispanic White.

### 3.2. Effect of the Diagnosis Group

The effect of the diagnosis group on MO_2_ was significant (*p* = 0.03; *η* = 0.44; OP = 0.67) in the AS group (Table 2), such that MO_2_ was lower in the NDR than in mild NPDR (*p* = 0.03). The effect of the diagnosis group on DO_2_ was marginally significant (*p* = 0.06; *η* = 0.37; OP = 0.55) in the AS group (Table 3), such that DO_2_ was lower in the NDR than in mild NPDR (*p* = 0.05). OEF was not significantly affected by the diagnosis group in all races (Table 4) (*p* ≥ 0.11).

### 3.3. Effect of Race

In the ND group, there was no significant effect of race on MO_2_ (*p* = 0.10) (Table 2). The effect of race on DO_2_ was significant (*p* = 0.04; *η* = 0.29; OP = 0.65) (Table 3), such that DO_2_ was marginally higher in HW than in NHW (*p* = 0.07). Race had a significant effect on OEF (*p* = 0.002; *η* = 0.47; OP = 0.95) (Table 4), such that OEF was higher in AS than in HW and NHW (*p* ≤ 0.006).

In the NDR group, there was no significant effect of race on MO_2_ (*p* = 0.12) (Table 2). DO_2_ was affected by race (*p* = 0.005; *η* = 0.28; OP = 0.89) (Table 3) and was higher in AA than in AS, HW, and NHW (*p* ≤ 0.03). There was no significant effect of race on OEF (*p* = 0.66) (Table 4).

In mild NPDR, there was a significant effect of race on MO_2_ (*p* = 0.04; *η* = 0.24; OP = 0.66) (Table 2), and MO_2_ was higher in AS than in NHW (*p* =0.03). DO_2_ was not significantly affected by race (*p* = 0.24) (Table 3). The effect of race on OEF was significant (*p* = 0.01; *η* = 0.30; OP = 0.80) (Table 4), such that OEF was higher in AS than in NHW (*p* = 0.02).

## 4. Discussion

Clinical evaluation of DR is based on visual examination and imaging of the retinal structures and vasculature. Given the variations in the prevalence of DR according to race, identification of race‐specific oxygen metabolic biomarkers may improve detection, progression, and treatment monitoring of DR. The present preliminary study with limited populations confirmed our hypothesis that there is an effect of race on retinal oxygen metabolic biomarkers in diabetic and ND subjects. Given the known effect of race on the prevalence and outcome of DR, the findings warrant future studies in larger cohorts to establish race‐specific biomarker threshold levels that can be applicable to a diverse patient population.

In the current study, no significant effect of the diagnosis group on DO_2_, MO_2_, and OEF was observed in AA, HW, and NHW without clinical DR or with mild DR. This finding is in agreement with earlier studies in Type 2 diabetics of all races, reporting reductions in DO_2_ and MO_2_ only in advanced stages of NPDR and PDR [32, 33]. In contrast, one study in European Whites with Type 1 diabetes showed a reduction in MO_2_ in no or mild DR compared to ND [31]. A previous study identified a larger central retinal vein with equivalent diameter as a risk factor in the incidence and progression of DR in northeastern Chinese individuals with Type 2 diabetes [40]. The reported increase in vessel diameter, which is a determinant of MO_2_ and DO_2_, is consistent with our finding of augmented MO_2_ and marginal elevation of DO_2_ in mild NPDR. Further studies in larger cohorts are warranted to establish the effect of the diagnosis group according to race.

Previous studies have shown that AS with diabetes were more likely to progress to severe DR than NHW diabetics [8], and that DR lesion distribution, an indicator of chance of progression to PDR, was higher in East AS than NHW and HW [41]. In the current study, with a relatively small sample size of subjects, we found that MO_2_ and OEF were higher in East AS than NHW with mild NPDR. This preliminary finding is suggestive of altered metabolic function, as also reflected in a previous study that showed an increase in MO_2_ in diabetic mice [42]. Furthermore, in AS without diabetes, OEF was elevated compared to HW and NHW. One potential explanation is a higher proportional demand for oxygen to maintain tissue health, as supported by a previous report of higher capillary density in Chinese adults compared to Caucasians [43]. Further studies in a larger AS cohort are needed to establish variations in MO_2_ and OEF in people with and without diabetes.

One study showed that AA with Type 2 diabetes were more likely to progress to DR at follow‐up than White people with diabetes (50% vs. 19%), even after adjusting for factors such as sex, HbA1c, blood pressure, and treatment [44]. The current preliminary study with a small sample size demonstrated that AA without clinical DR had a higher DO_2_ than people of the other studied racial groups. In moderate to severe NPDR, another study found increased SO_2V_ in the AA population [45]. Together, these studies imply potential variations in the compensatory responses to maintain metabolic function prior to the development and at more advanced stages of DR. Notably, given the small sample size in the AA group, additional studies are needed to confirm these preliminary findings.

The prevalence of diabetes and DR has been shown to be higher in HW than NHW (European Whites) in the United States [6, 15, 46]. In the current preliminary study, in subjects without diabetes, DO_2_ was marginally higher in HW compared to NHW due to elevated blood flow. These preliminary results warrant further studies in a larger cohort to investigate the potential of a reduction in blood flow compensatory capacity under diabetic conditions that may contribute to the observed increased prevalence of DR in HW.

There are several limitations to this study. First, in this pilot study, race was treated as a biological variable. However, interpretation of racial differences in the biomarkers requires consideration of race as a social construct with complex genetic, environmental, socioeconomic, and healthcare access components. Specifically, confounding factors such as healthcare or systemic inequities that disproportionately affect certain racial groups as well as differences in environmental exposures and medical comorbidity burden also contributed to the reported differences. Second, this preliminary study had an unequal distribution of subjects within racial groups and a small sample size, which may have limited the comparison of some differences between groups. Future studies in larger and more balanced cohorts are needed to confirm the findings and establish baselines for retinal oxygen metabolic biomarkers according to race. Third, although the method employed in the current study corrected for variations in retinal pigmentation, their contribution to the results may not have been eliminated completely. In addition, covariates such as hypertension, hyperlipidemia, duration of diabetes, body mass index, smoking, medication use, and social determinants of health were not accounted for in the statistical models, warranting further comprehensive studies.

## 5. Conclusion

In conclusion, the current preliminary study demonstrated for the first time associations of race with retinal oxygen metabolic biomarkers in NDs and diabetics at early stages of retinopathy. The findings underscore the importance of considering racial differences for detecting, monitoring progression, and guiding treatment of DR. Future research that includes a larger cohort and at all stages of DR, with the inclusion of potential confounding factors, is warranted to establish race‐specific retinal oxygen metabolic biomarkers for the clinical management of DR.

## Funding

This work was supported by the National Eye Institute, Bethesda, MD (EY030115 and EY029220) and an unrestricted departmental award from Research to Prevent Blindness, New York, NY.

## Conflicts of Interest

The authors declare no conflicts of interest.

## Data Availability

The data that support the findings of this study are available from the corresponding author upon reasonable request.

## References

[1] Lundeen E. A. , Burke-Conte Z. , Rein D. B et al., Prevalence of Diabetic Retinopathy in the US in 2021, JAMA Ophthalmol. (2023) 141, no. 8, 10.1001/jamaophthalmol.2023.2289. PMC10273133 37318810

[2] Wang W. and Lo A. C. Y. , Diabetic Retinopathy: Pathophysiology and Treatments, International Journal of Molecular Sciences. (2018) 19, no. 6, 10.3390/ijms19061816, 2-s2.0-85048890879. PMC6032159 29925789

[3] Meyerle C. B. , Chew E. Y. , and Ferris F. L. , Nonproliferative Diabetic Retinopathy, 2008, Humana Press, Totowa, NJ.

[4] Stefánsson E. , Chan Y. K. , Bek T. , Hardarson S. H. , Wong D. , and Wilson D. I. , Laws of Physics Help Explain Capillary Non-Perfusion in Diabetic Retinopathy, Eye. (2018) 32, no. 2, 210–212, 10.1038/eye.2017.313, 2-s2.0-85041842683. 29350688 PMC5811745

[5] Singh R. , Ramasamy K. , Abraham C. , Gupta V. , and Gupta A. , Diabetic Retinopathy: an Update, Indian Journal of Ophthalmology. (2008) 56, no. 3, 179–188, 10.4103/0301-4738.40355, 2-s2.0-42549115245. PMC2636123 18417817

[6] Cheng Y. J. , Kanaya A. M. , Araneta M. R. G et al., Prevalence of Diabetes by Race and Ethnicity in the United States, 2011-2016, JAMA. (2019) 322, no. 24, 2389–2398, 10.1001/jama.2019.19365. 31860047 PMC6990660

[7] Herman W. H. , Ma Y. , Uwaifo G et al., Differences in A1C by Race and Ethnicity Among Patients with Impaired Glucose Tolerance in the Diabetes Prevention Program, Diabetes Care. (2007) 30, no. 10, 2453–2457, 10.2337/dc06-2003, 2-s2.0-35148859728. 17536077 PMC2373980

[8] Pardhan S. , Gilchrist J. , and Mahomed I. , Impact of Age and Duration on sight-threatening Retinopathy in South Asians and Caucasians Attending a Diabetic Clinic, Eye. (2004) 18, no. 3, 233–240, 10.1038/sj.eye.6700629, 2-s2.0-1842430897. 15004570

[9] Baker R. S. , Diabetic Retinopathy in African Americans: Vision Impairment, Prevalence, Incidence, and Risk Factors, International Ophthalmology Clinics. (2003) 43, no. 4, 105–122, 10.1097/00004397-200343040-00011. 14574205

[10] Roy M. S. , Diabetic Retinopathy in African Americans with Type 1 Diabetes: the New Jersey 725: I. Methodology, Population, Frequency of Retinopathy, and Visual Impairment, Archives of Ophthalmology. (2000) 118, no. 1, 97–104, 10.1001/archopht.118.1.97, 2-s2.0-0033989403. 10636422

[11] Haw J. S. , Shah M. , Turbow S. , Egeolu M. , and Umpierrez G. , Diabetes Complications in Racial and Ethnic Minority Populations in the USA, Current Diabetes Reports. (2021) 21, no. 1, 10.1007/s11892-020-01369-x. PMC7935471 33420878

[12] Coney J. M. and Scott A. W. , Racial Disparities in the Screening and Treatment of Diabetic Retinopathy, Journal of the National Medical Association. (2022) 114, no. 2, 171–181, 10.1016/j.jnma.2021.12.011. 35105457

[13] Maturi J. , Maturi V. , Scott A. W. , Carson K. A. , Ciulla T. et al., Effect of Race and Insurance Status on Treatment and Outcomes in Diabetic Retinopathy: Analysis of 43 274 Eyes Using the IRIS Registry, J Vitreoretin Dis. (2024) 8, no. 3, 270–279, 10.1177/24741264231221607. 38770080 PMC11102718

[14] Munoz B. , West S. K. , Rubin G. S. , Schein O. D. , Quigley H. A. et al., Causes of Blindness and Visual Impairment in a Population of Older Americans: the Salisbury Eye Evaluation Study, Archives of Ophthalmology. (2000) 118, no. 6, 819–825, 10.1001/archopht.118.6.819, 2-s2.0-0344002670. 10865321

[15] Wong T. Y. , Klein R. , Islam F. M et al., Diabetic Retinopathy in a multi-ethnic Cohort in the United States, American Journal of Ophthalmology. (2006) 141, no. 3, 446–455, 10.1016/j.ajo.2005.08.063, 2-s2.0-33144486463. 16490489 PMC2246042

[16] Sanjiv N. , Osathanugrah P. , Harrell M et al., Race and Ethnic Representation Among Clinical Trials for Diabetic Retinopathy and Diabetic Macular Edema Within the United States: a Review, Journal of the National Medical Association. (2022) 114, no. 2, 123–140, 10.1016/j.jnma.2021.12.016. 35078668

[17] Hirsch K. , Cubbidge R. P. , and Heitmar R. , Dual Wavelength Retinal Vessel Oximetry-Influence of Fundus Pigmentation, Eye. (2023) 37, no. 11, 2246–2251, 10.1038/s41433-022-02325-7. 36460856 PMC9716545

[18] Blair N. P. , Wanek J. , Felder A. E et al., Retinal Oximetry and Vessel Diameter Measurements with a Commercially Available Scanning Laser Ophthalmoscope in Diabetic Retinopathy, Investigative Ophthalmology & Visual Science. (2017) 58, no. 12, 10.1167/iovs.17-21934, 2-s2.0-85032565925. PMC5656420 29079858

[19] Bisignano K. K. , Smith J. D. , and Harrison W. W. , Variations in Retinal Oxygen Saturation in a Diverse Healthy Population, Clinical Optometry. (2024) 16, 147–155, 10.2147/opto.s468076. 39045010 PMC11265219

[20] Aref A. A. , Maleki S. , Tan O. , Huang D. , Varma R. , and Shahidi M. , Relating Glaucomatous Visual Field Loss to Retinal Oxygen Delivery and Metabolism, Acta Ophthalmologica. (2019) 97, no. 7, e968–e972, 10.1111/aos.14120, 2-s2.0-85064839914. 31016869

[21] Bata A. M. , Fondi K. , Szegedi S et al., Age-Related Decline of Retinal Oxygen Extraction in Healthy Subjects, Investigative Ophthalmology & Visual Science. (2019) 60, no. 8, 3162–3169, 10.1167/iovs.18-26234, 2-s2.0-85070440641. 31335953

[22] Kallab M. , Hommer N. , Schlatter A et al., Retinal Oxygen Metabolism and Haemodynamics in Patients with Multiple Sclerosis and History of Optic Neuritis, Frontiers in Neuroscience. (2021) 15, 10.3389/fnins.2021.761654. PMC8546107 34712117

[23] Shahidi M. , Felder A. E. , Tan O. , Blair N. P. , and Huang D. , Retinal Oxygen Delivery and Metabolism in Healthy and Sickle Cell Retinopathy Subjects, Investigative Ophthalmology & Visual Science. (2018) 59, no. 5, 1905–1909, 10.1167/iovs.17-23647, 2-s2.0-85045103226. 29677351 PMC5886143

[24] Werkmeister R. M. , Schmidl D. , Aschinger G et al., Retinal Oxygen Extraction in Humans, Scientific Reports. (2015) 5, no. 1, 10.1038/srep15763, 2-s2.0-84946078381. PMC4621499 26503332

[25] Felder A. E. , Wanek J. , Blair N. P. , and Shahidi M. , Inner Retinal Oxygen Extraction Fraction in Response to Light Flicker Stimulation in Humans, Investigative Ophthalmology & Visual Science. (2015) 56, no. 11, 10.1167/iovs.15-17321, 2-s2.0-84945208100. PMC4611954 26469748

[26] Tayyari F. , Khuu L.-A. , Flanagan J. G. , Singer S. , Brent M. H. , and Hudson C. , Retinal Blood Flow and Retinal Blood Oxygen Saturation in Mild to Moderate Diabetic Retinopathy, Investigative Ophthalmology & Visual Science. (2015) 56, no. 11, 6796–6800, 10.1167/iovs.15-17481, 2-s2.0-84945137186. 26567792

[27] Palkovits S. , Lasta M. , Told R et al., Retinal Oxygen Metabolism During Normoxia and Hyperoxia in Healthy Subjects, Investigative Ophthalmology & Visual Science. (2014) 55, no. 8, 4707–4713, 10.1167/iovs.14-14593, 2-s2.0-84905234225. 25015353

[28] Song W. , Wei Q. , Liu W et al., A Combined Method to Quantify the Retinal Metabolic Rate of Oxygen Using Photoacoustic Ophthalmoscopy and Optical Coherence Tomography, Scientific Reports. (2014) 4, no. 1, 10.1038/srep06525, 2-s2.0-84923270736. PMC4185377 25283870

[29] Yi J. , Liu W. , Chen S et al., Visible Light Optical Coherence Tomography Measures Retinal Oxygen Metabolic Response to Systemic Oxygenation, Light: Science & Applications. (2015) 4, no. 9, 10.1038/lsa.2015.107, 2-s2.0-84989245372. PMC4674267 26658555

[30] Pai V. , Janku P. , Lindner T et al., A New Approach to Retinal Oxygen Extraction Measurement Based on Laser Speckle Flowgraphy and Retinal Oximetry, Translational Vision Science & Technology. (2024) 13, no. 12, 10.1167/tvst.13.12.12. PMC11636657 39661379

[31] Fondi K. , Wozniak P. A. , Howorka K et al., Retinal Oxygen Extraction in Individuals with Type 1 Diabetes with No or Mild Diabetic Retinopathy, Diabetologia. (2017) 60, no. 8, 1534–1540, 10.1007/s00125-017-4309-0, 2-s2.0-85019705861. 28547132 PMC5491565

[32] Hommer N. , Kallab M. , Schlatter A et al., Retinal Oxygen Metabolism in Patients with Type 2 Diabetes and Different Stages of Diabetic Retinopathy, Diabetes. (2022) 71, no. 12, 2677–2684, 10.2337/db22-0219. 36107468 PMC9862478

[33] Rahimi M. , Hossain F. , Leahy S. , Blair N. P. , Jiang X. , and Shahidi M. , Inner Retinal Oxygen Delivery and Metabolism in Progressive Stages of Diabetic Retinopathy, Scientific Reports. (2024) 14, no. 1, 10.1038/s41598-024-54701-w. PMC10883954 38388657

[34] Felder A. E. , Wanek J. , Blair N. P et al., The Effects of Diabetic Retinopathy Stage and Light Flicker on Inner Retinal Oxygen Extraction Fraction, Investigative Ophthalmology & Visual Science. (2016) 57, no. 13, 5586–5592, 10.1167/iovs.16-20048, 2-s2.0-84992359672. 27768785 PMC6016433

[35] Rahimi M. , Kashani A. H. , Blair N. P. , and Shahidi M. , Alterations in Retinal Vascular and Oxygen Metrics in Treated and Untreated Proliferative Diabetic Retinopathy: a Case Report, Case Reports in Ophthalmology. (2022) 13, no. 3, 686–691, 10.1159/000526569. 36845454 PMC9944214

[36] Wilkinson C. P. , Ferris F. L. , Davis M et al., Proposed International Clinical Diabetic Retinopathy and Diabetic Macular Edema Disease Severity Scales, Ophthalmology. (2003) 110, no. 9, 1677–1682, 10.1016/s0161-6420(03)00475-5, 2-s2.0-0042932653. 13129861

[37] Tan O. , Liu G. , Liang L et al., En Face Doppler Total Retinal Blood Flow Measurement with 70 Khz Spectral Optical Coherence Tomography, Journal of Biomedical Optics. (2015) 20, no. 6, 10.1117/1.jbo.20.6.066004, 2-s2.0-84935889745. PMC4462711 26062663

[38] Hammer M. , Vilser W. , Riemer T. , and Schweitzer D. , Retinal Vessel oximetry-calibration, Compensation for Vessel Diameter and Fundus Pigmentation, and Reproducibility, Journal of Biomedical Optics. (2008) 13, no. 5, 10.1117/1.2976032, 2-s2.0-51349099299. 19021395

[39] West J. B. , Pulmonary Physiology and Pathophysiology: An Integrated, Case-based Approach, 2007, Lippincott Williams & Wilkins.

[40] Lin Z. , Wen L. , Wang Y. , Li D. , Zhai G. et al., Incidence, Progression and Regression of Diabetic Retinopathy in a Northeastern Chinese Population, British Journal of Ophthalmology. (2022) . 10.1136/bjo-2022-321384 35864776

[41] He Y. , Verma A. , Nittala M. G et al., Ethnic Variation in Diabetic Retinopathy Lesion Distribution on Ultra-widefield Imaging, American Journal of Ophthalmology. (2023) 247, 61–69, 10.1016/j.ajo.2022.10.023. 36368347

[42] Liu W. , Wang S. , Soetikno B et al., Increased Retinal Oxygen Metabolism Precedes Microvascular Alterations in Type 1 Diabetic Mice, Investigative Ophthalmology & Visual Science. (2017) 58, no. 2, 981–989, 10.1167/iovs.16-20600, 2-s2.0-85012293335. 28535269 PMC5308771

[43] Bujor I. , Chua J. , Tan B et al., Comparing Optical Coherence Tomography Angiography Metrics in Healthy Chinese and Caucasian Adults, Journal of Personalized Medicine. (2024) 14, no. 8, 10.3390/jpm14080834. PMC11355270 39202025

[44] Harris E. L. , Sherman S. H. , and Georgopoulos A. , Black-White Differences in Risk of Developing Retinopathy Among Individuals with Type 2 Diabetes, Diabetes Care. (1999) 22, no. 5, 779–783, 10.2337/diacare.22.5.779, 2-s2.0-0032945301. 10332681

[45] Garvey S. L. , Khansari M. M. , Jiang X. , Varma R. , and Shahidi M. , Assessment of Retinal Vascular Oxygenation and Morphology at Stages of Diabetic Retinopathy in African Americans, BMC Ophthalmology. (2020) 20, no. 1, 10.1186/s12886-020-01566-y. PMC7368779 32682412

[46] Kempen J. H. , O’Colmain B. J. , Leske M. C , et al., Eye Diseases Prevalence Research Group , The Prevalence of Diabetic Retinopathy Among Adults in the United States, Archives of Ophthalmology. (2004) 122, no. 4, 552–563, 10.1001/archopht.122.4.552, 2-s2.0-11144356305. 15078674

